# Health Inequality between Migrant and Non-Migrant Workers in an Industrial Zone of Vietnam

**DOI:** 10.3390/ijerph16091502

**Published:** 2019-04-28

**Authors:** Kiet Tuan Huy Pham, Long Hoang Nguyen, Quan-Hoang Vuong, Manh-Tung Ho, Thu-Trang Vuong, Hong-Kong T. Nguyen, Giang Thu Vu, Huong Lan Thi Nguyen, Bach Xuan Tran, Carl A. Latkin, Cyrus S. H. Ho, Roger C. M. Ho

**Affiliations:** 1 Institute for Preventive Medicine and Public Health, Hanoi Medical University, Hanoi 100000, Vietnam; phamhuytuankiet_vkt@fpt.vn (K.T.H.P.); bach.ipmph@gmail.com (B.X.T.); 2 Center of Excellence in Behavioral Medicine, Nguyen Tat Thanh University, Ho Chi Minh City 700000, Vietnam; longnh.ph@gmail.com (L.H.N.); pcmrhcm@nus.edu.sg (R.C.M.H.); 3 Centre for Interdisciplinary Social Research, Phenikaa University, Yen Nghia, Ha Dong, Hanoi 100803, Vietnam; hoang.vuongquan@phenikaa-uni.edu.vn (Q.-H.V.); tung.homanh@phenikaa-uni.edu.vn (M.-T.H.); 4 Faculty of Economics and Finance, Phenikaa University, Yen Nghia, Ha Dong, Hanoi 100803, Vietnam; 5 Sciences Po Paris, Campus de Dijon, 21000 Dijon, France; thutrang.vuong@sciencespo.fr; 6 A.I. for Social Data Lab (AISDL), Vuong & Associates, 3/161 Thinh Quang, Dong Da district, Hanoi 100000, Vietnam; htn2107@caa.columbia.edu; 7 Center of Excellence in Evidence-based Medicine, Nguyen Tat Thanh University, Ho Chi Minh City 700000, Vietnam; giang.coentt@gmail.com; 8 Institute for Global Health Innovations, Duy Tan University, Danang 550000, Vietnam; 9 Bloomberg School of Public Health, Johns Hopkins University, Baltimore, MD 21205, USA; carl.latkin@jhu.edu; 10 Department of Psychological Medicine, National University Hospital, Singapore 119074, Singapore; cyrushosh@gmail.com; 11 Department of Psychological Medicine, Yong Loo Lin School of Medicine, National University of Singapore, Singapore 119228, Singapore; 12 Biomedical Global Institute of Healthcare Research & Technology (BIGHEART), National University of Singapore, Singapore 119228, Singapore

**Keywords:** inequality, health status, health-related quality of life, migration, industry, worker

## Abstract

Vietnam has experienced massive internal migration waves from rural to industrialized zones. However, little efforts have been made to understand differences in health conditions and health-related quality of life (HRQOL) between local and migrant industrial workers. This study aimed to examine the inequality in health status and HRQOL between these workers. We conducted a cross-sectional study of 289 Vietnamese workers at three industrial areas in Hanoi and Bac Ninh. Self-reported health status and HRQOL were measured using the EuroQOL-5 dimensions-5 levels (EQ-5D-5L) instrument. Sociodemographic, working, and environmental factors were also investigated. Overall, the mean EQ-5D index was 0.74 (SD = 0.21) and the average number of health problems in the last 12 months in our sample was 1.91 (SD = 1.63) problems. Migrant people had a lower EQ-5D index (β = −0.08, *p* < 0.01) and more health problems (β = 0.20, *p* < 0.05) compared to local workers. Those being male, working in the same posture more than 60 min, and exposed to more hazards at work were correlated with a lower EQ-5D index and higher number of health problems. The results highlighted inequalities in health status and HRQOL between migrant and local workers. Reinforcing regular health check-ups, ensuring sufficient protective equipment and working conditions may help improve the health outcomes of the workers.

## 1. Introduction

The Sustainable Development Goals by 2030 underline the need to alleviate inequality in society (Goal 10) as well as ensure the healthy lives for all individuals (Goal 3) [1]. However, migrant workers worldwide are among disadvantaged groups who have suffered substantial health inequality [2]. Prior studies reported that migrant workers are often exposed to hazardous occupational factors; had long working hours, insecure employment, and unstable living conditions; and were at high risk of injuries and even death [3,4,5,6]. In addition, victims of workplace injustice or those facing more adverse health outcomes in the workplace tend to be the marginalized migrants [7,8,9]. Previous literature indicates that migrant workers are more likely to be socially isolated due to cultural differences, have a poor living standard, live in crowded housing with a deficit of clean water and sanitation, receive poor health care access, as well as highly engage in risk behaviors such as substance misuse and unsafe sexual practices [3,4,10,11]. These unfavorable conditions trigger a reduction of work productivity and economic growth, as well as a threat to public health and social stability in communities where migrant workers live [6]. This challenge requires significant efforts to address the unmet need and assure equal rights, including health outcomes and health care access, of migrant workers with the local counterparts. 

However, tracking health inequality between migrant workers and their local counterparts are still constrained. This limitation may be due to the lack of surveillance systems [12]. Workplace health data rely on reporting from regular health examination, but available evidence points out a systematic exclusion of data from underserved workers, known as “filtering out,” either due to a failure to report or capture a work-related health outcome [12,13]. In the case that clinical and laboratory indicators are difficult to be measured, self-reported health outcomes should be applied for monitoring the interventions’ progress. Health-related quality of life (HRQOL), which refers to the perception and preference of individuals to their physical, psychological, and social conditions [14], are particularly useful. The concept of HRQOL is broad and covers multiple perspectives instead of only health conditions as clinical measures do [14,15]. In addition, self-reported HRQOL is well-recognized as an indicator to predict morbidity and mortality [16,17,18]. Evaluating HRQOL has implications for decision makers as it provides a simple, low-cost tool for rapid assessment and regular monitoring. Globally, HRQOL in migrant workers have been investigated and found to be associated with socio-economic characteristics (marital status, employment duration) [19], mental health (work-related stress or depression) [20], morbidities [5,20], or health service utilization [19].

Vietnam has undergone rapid industrialization since economic reforms in 1986, leading to massive waves of labor mobility and internal worker migration from rural areas to places where manufacturers are located. This labor mobility helps millions of people moving out of poverty and has contributed considerably to the economic growth of Vietnam in the past three decades [9,21,22]. A report from the United Nations Industrial Development Organization in 2015 revealed that in Vietnam, more than 2.3 million people worked in industrial zones, and 70% of them were migrant workers with a predominance of females and youth [23]. However, Quynh et al. found that most of them rented accommodation with very poor quality and sanitation standards [24]. The extant literature on the relationship between health and internal labor migration in Vietnam has primarily examined a number of prevalent health risks, such as HIV/AIDS vulnerability and transmission [25,26,27], reproductive tract infection among female migrants [28,29], excessive alcohol consumption among male street laborers [30], sexual practices and sexually transmissible infections among migrant sex workers [31,32], or malaria incidence in the at-risk migrant population [33]. Given continuing migration and uneven economic integration and growth, ensuring the detection of health issues and timely provision of health services in migrant workers is crucial to national health equity [34]. The study aims to bring attention to the inequalities in health status and health-related quality of life between the migrant and local industrial workers in Vietnam.

## 2. Materials and Methods

### 2.1. Study Design and Participant Recruitment

Data of this study were derived from a cross-sectional study on Vietnamese workers at three industrial areas in Hanoi and Bac Ninh—the most developed industrial zones in the North of Vietnam. We approached participants via a health communication session in each industrial plant organized by the Hanoi Medical University. They were conveniently invited to participate in the study if they had the following eligibility criteria: (1) Being 18 years old or above; (2) working under labor contracts in the three aforementioned plants; (3) working at the factory for six months or more; (4) agreeing to participate in the study; (5) being able to communicate with the interviewers without any language or health barriers. Participants who suffered from serious illness were excluded from the recruitment process. 

In this study, we applied a formula for testing two population proportions (two-sided test) in order to calculate the sample size. With level of significance = 5%, power of the test = 90%, expected prevalence of health problems among native workers = 50% and among migrant workers = 70%, the sample size was 124 workers per group, thus 248 workers should be recruited into the study. Indeed, we invited all workers who met our inclusion criteria and participated in the health communication sessions at the three industrial plants. There were 289 workers invited and agreed to participate in the study, and data of 280 workers (response rate 96.9%) were eligible for data analysis (137 native workers and 143 migrant workers). This study protocol was approved by the Institutional Review Board of Youth Research Institute (01a QĐ/VNCTN).

### 2.2. Measures and Instrument

Data collection was performed via 20-min face-to-face interviews using a structured questionnaire. The questionnaire was first piloted among 20 industrial workers to ensure the text, language, and logical order of items. This questionnaire was finalized after a minor revision. In the field, workers were introduced to the purpose of the study, the benefits, and the drawbacks in joining the study. If they agreed to participate in the study, they were invited to go to a small private counseling room at the factory to protect the confidentiality of the participants. No factory staff were involved in the recruitment process or the conduct of the study. 

#### 2.2.1. Primary Outcomes

We asked workers to report their current health problems (diseases or symptoms) that they were suffering from at the time of the interview. Each disease or symptom they listed was considered one health problem. We also measured health-related quality of life (HRQOL) of workers by employing the EuroQol-5 dimensions-5 levels (EQ-5D-5L) instrument [35]. This tool has been validated previously in Vietnam among the general population and HIV/AIDS people [17,36]. This tool measures five dimensions of health-related quality of life including mobility, self-care, usual activities, pain/discomfort, and anxiety/ depression with five response levels: From no problems (code 1) to extreme problems (code 5) [37]. Together, they can be combined to profile a health state of each individual, which ranges from 11111 (full health) to 55555 (the worst health) [17]. Each health state can be transformed to a single EQ-5D utility index by using a Thailand cross-walk value set [35]. This value set had a score range from 1 (for health state 11111) to -0.452 (for health state 55555). Moreover, participants reporting “no problems” were classified into “No problem” group; otherwise, they were classified into the “having problem” group. The Cronbach’s alpha of EQ-5D-5L was good at 0.8183.

#### 2.2.2. Other Covariates

Socioeconomic characteristics and Immigration status: We asked participants to report their information about gender, age, education attainment, marital status, monthly income, whether they lived with their family or not. We also collected information regarding migration, including whether they were a migrant or not, duration of migration, and the distance from their homeland to their factories. According to the International Organization for Migration, the term “migrant” was defined as “any person who is moving or has moved across an international border or within a State away from his/her habitual place of residence” [38]. 

Working environment characteristics: Participants were asked to estimate their working years, average number of working hours per day, toxic factors that they were exposured during working (including noise, dust, high temperature, toxic gas, poor lighting, high humidity, toxic chemicals, and working environment with high risk of accidence), how long they were in a constant posture when working (less than 30 min/30–60 min/more than 60 min). 

Soical support: We asked workers to express their attitude towards social support and community cohesion around their current living area (including neighbourhood cohesion [39], neighbourhood disorder [40], and social disorder [40]). In particular, they were asked the following statements:

1) People in this area are willing to help each other (Yes = 1 point, No = 0 point)
2) People around living place are willing to help neighbors (Yes = 1 point, No = 0 point)
3) People live in harmony (Yes = 1 point, No = 0 point)
4) People around can be trusted and reliable (Yes = 1 point, No = 0 point)
5) People share the same value and life concept (Yes = 1 point, No = 0 point)
6) This area has a tremendous amount of trash (No = 1 point, Yes = 0 point)
7) This area has society’s vices (No = 1 point, Yes = 0 point)
8) This area has many fights and quarrels (No = 1 point, Yes = 0 point)

The total social support score ranged from 0 to 8 points. The internal consistency reliability was acceptable with the Cronbach’s alpha = 0.6357.

Alcohol and tobacco use behavior: We screened participants’ hazardous drinking practices by applying the Alcohol Use Disorders Identification Test-Consumption (AUDIT-C). This instrument has three questions including 1) “How often do you have a drink containing alcohol?”; 2) “How many standard drinks containing alcohol do you have on a typical day?”; and 3) “How often do you have six or more drinks on one occasion?” [41]. Each question has five responses with a score range from 0 to 4, and the total score is from 0 to 12 [41]. Male workers with four points or more and female workers with three points or more were grouped in the “hazardous drinking” group. We also asked participants to report their current smoking status in the last 30 days. They were classified in the “current smoking” group if they answered “Yes” for the question “have you smoked a cigarette in the last 30 days?”. 

### 2.3. Statistical Analysis

Data analysis was done in STATA version 12 (Stata Corp. LP, College Station, TX, USA). Demographic characteristics of participants, as well as career characteristics, social support, HRQOL, and health risk behavior were compared between migrant and local workers by using a Chi-squared test and Mann-whitney test. Generalized linear models (GLM) were conducted to identify associated factors with HRQOL (model 1, identity, Gaussian) and a number of health problems of respondents (model 2, log, Poisson). A *p*-value of less than 0.05 was considered statistically significant. 

## 3. Results

Table 1 shows that most of the workers were female (82.8%), had a high school education or above (91.7%), and had a spouse/partner (95.7%). The mean age of the participants was 31.9 years (SD = 4.5), and their average annual income was 272.4 USD (SD = 96.7). Among migrant workers, the mean distance from their homelands to the factories was 86.3 kilometers (SD = 95.6), and the average duration of migration was 2.1 years (SD = 3.0). No difference was found between migrant and non-migrant workers regarding gender, education, marital status, income, smoking, and alcohol drink behaviors (*p* > 0.05). Non-migrant workers had a significantly higher age (mean = 32.8 years, SD = 4.8) than that of migrant workers (mean = 31.0 years, SD = 4.0). More than a half of non-migrant workers (55.2%) lived with parents, compared to 39.4% of migrant workers (*p* < 0.01). 

Table 2 describes that more than half of the participants had more than 60 min working in one posture. A high proportion of participants were exposed to noise (77.8%), high temperature (58.7%), and dust (50.7%). The mean number of health hazards that a worker was exposed to was 2.8 (SD = 2.1). In regards to social environmental factors, more than 50% of the participants believed that people in their living area were willing to help each other (57.3%), neighbors (55.4%), and lived in harmony (56.5%). The mean social support score was 4.5 (SD = 2.1). A higher proportion of migrant workers were exposed to noise (83.1%) compared to non-migrant workers (71.9%) (*p* = 0.03). Meanwhile, non-migrant workers had higher years of working (mean = 10.3 years, SD = 3.4) than migrant workers (mean = 9.5 years, SD = 4.0) (*p* = 0.01). Otherwise, there was no difference between migrant and non-migrant workers regarding time for working in one posture, working hours per day, number of hazardous occupational exposures, and social support score (*p* > 0.05).

Table 3 shows that in all categories of health problems, except for usual activity, there were more migrant workers than their non-migrant counterparts. There were more migrants having at least one health problem than the local workers (*p* = 0.045). There were 61.0% of the participants who experienced pain/discomfort (52.4% in non-migrant workers and 69.2% in migrant workers, *p* = 0.01), 61.7% feeling anxiety/depression (52.0% in non-migrant workers and 70.7% in migrant workers, *p* < 0.01) and 30.6% had problems with their usual activity (23.6% in non-migrant workers and 37.0% in migrant workers, *p* = 0.02). The mean EQ-5D index was 0.74 (SD = 0.21) and the average number of health problems in our sample was 1.91 (SD = 1.63) problems. The difference of EQ-5D index between migrant (mean = 0.69, SD = 0.19) and non-migrant (mean = 0.78, SD = 0.23) workers was statistically significant (*p* < 0.01).

Table 4 indicates that after adjusting for socio-economic, social, and behavioral factors, the results show that migrant people were more likely to have a lower EQ-5D index (Coef.= −0.08, *p*-value <0.01) compared to local individuals, and were also more likely to have more health problems (Coef. = 0.20, *p*-value <0.05). 

Female workers were found to have a higher EQ-5D index than their male counterparts. Individuals working in one posture for more than 60 min were likely to have more health problems (Coef. = 0.39, *p*-value <0.01). A number of health hazards exposure at work had a significant negative correlation with the EQ5D5L index (Coef. = −0.02; *p* < 0.05) and a small positive association with the number of health problems (Coef. = 0.14; *p*-value <0.01). 

## 4. Discussion

This is one of the first studies to investigate the inequality in health conditions and HRQOL between migrant and native industrial workers in Vietnam. Our findings indicated a significantly lower HRQOL and a higher number of health issues in the migrants compared to their local counterparts. Moreover, our results revealed some contributors to health and HRQOL impairments, which could be useful for further interventions to improve the occupational health of both local and migrant industrial workers in Vietnam.

HRQOL is an individual’s subjective perception of physical, psychological, and social conditions [14], which are determined by various factors; namely socio-economic and employment characteristics, morbidities, behavior, and social support [17,20,42]. In this study, after adjusting for these potential confounders, our finding enriches current literature that migrant workers had poorer HRQOL and health conditions than local-born workers. Indeed, migrant workers in our study reported higher rates of having problems in all five EQ-5D domains than their local counterparts, which resulted in a lower HRQOL index. Moreover, this finding can also be explained by the presence of morbidities, which is an important driver for HRQOL [5]. Likewise, migrant workers had a higher likelihood of reporting health issues than local workers. Our study aligns with prior works in China, indicating significantly lower HRQOL among rural-to-urban migrant workers than the general population [5,20] as well as urban residents [43]. However, our finding was different from a study of Hesketh et al., which found that migrant workers had better health status than local workers who had similar socio-economic and employment background [6]. The authors called this phenomenon the “healthy migrant effect,” and explained by the selection bias—that only the healthier members of a population migrated and were recruited into the study [6]. Nonetheless, in this study, even with similar socio-economic status, social support, occupational working environment, and behavior, migrant workers’ health condition and HRQOL were not equal to local workers’ health. This implies a significant inequality between migrant and local workers, suggesting further interventions should be warranted to tackle this gap.

Our current study indicated a remarkably lower HRQOL of both worker groups compared to the general Vietnamese population (mean = 0.91) [17]. This can be understood to mean that industrial workers are exposed to many hazardous, work in a heavy pressure atmosphere, and have heavy work-loads, which perhaps deteriorate their physical and psychological health significantly, resulting in HRQOL impairment. In fact, more than two third of our respondents experienced problems in pain/discomfort and anxiety/depression, while only 10 to 50 percent of the general population suffered these issues [17]. The finding also indicated that the duration of working in a fixed posture was associated with HRQOL and a number of health problems. In particular, working in that condition longer than 60 min was associated with an increase in the chance of having more health problems. Moreover, those experiencing more hazardous occupational factors (noise, dust, high temperature, etc.) had both a lower HRQOL and a higher number of health issues. Our results were in line with prior studies in the world, which revealed the close connection between the high level of adverse condition exposures and HRQOL impairment [20,44]. Workers’ right associations and policy-makers can target this factor to improve the health outcome of industrial workers.

There are several implications that have been drawn from this study. First, educational programs to promote the social responsibility of employers in ensuring health and HRQOL of workers, especially migrant workers. Local authorities and employers should facilitate and support the use of occupational health service and regular health examination among migrant workers [45,46], enabling them to increase their self-protection awareness of their health and HRQOL. Second, employers should also take into account ways to reduce work that requires remaining in one posture for a long time, as well as providing sufficient protective equipment to avoid the adverse effects of hazardous occupational exposures. Third, regular monitoring of health status and HRQOL of factory workers, particularly migrant individuals, by using a simple tool such as the EQ-5D-5L as well as performing regular health screening should be mandated and regulated by the government, which can help workers and managers to detect the health problems and design timely interventions to improve health conditions and the HRQOL of workers.

Our strength is the use of validated international measures such as the EQ-5D-5L, and AUDIT-C, which enabled us to compare the findings with other studies in the world. However, the current study has several limitations. First, it uses a convenience sample rather than a national census. The sample was drawn primarily from three industrial zones in two Northern provinces, which might not reflect inequalities in health status between migrant and non-migrant workers in other factories or industrial zones in Vietnam. Follow-up studies should be conducted in other provinces to confirm the consistency of results, in addition to enlarging the sample size to assess the extent of the health inequalities. Moreover, given that different foreign investors have different working cultures, such that workers may have longer or shorter resting time depending on the regulations of their foreign-invested factories, the results here should not be generalized. Second, the analysis of the study relies on the actual self-reported health status by the participants. It is necessary to further study the data of self-rating health indicators and objective health measures. The results of the statistical analysis should be interpreted with caution. Last but not least, this study was a cross-sectional study; future research should include the comparison of the health status and quality of life of migrant workers over time.

## 5. Conclusions

The results highlighted inequalities in health status and HRQOL between migrant and local workers. Cooperating policies that focus on regular health examination of workers, social responsibility of employers, providing sufficient protective equipment, and allowing workers to change their posture more often will help improve the health outcomes of the workers.

## Figures and Tables

**Table 1:** Socio-economic status of respondents.

Characteristics	Non-Migrant		Migrant		Total		*p*-Value
	*n*	%	*n*	%	*n*	%	
Total	137	48.9	143	51.1	280	100	
Gender							
Male	28	20.6	20	14.0	48	17.2	0.14 ^a^
Female	108	79.4	123	86.0	231	82.8	
Education							
Under High school	14	10.2	9	6.4	23	8.3	0.47 ^a^
High school	81	59.1	90	63.8	171	61.5	
Above High school	42	30.7	42	29.8	84	30.2	
Marital status							
Single	5	3.7	7	4.9	12	4.3	0.61 ^a^
Having spouse/partner	132	96.4	136	95.1	268	95.7	
Currently living with							
Parents	75	55.2	56	39.4	131	47.1	<0.01 ^a^
Spouse	110	80.9	115	81.0	225	80.9	0.98 ^a^
Children	87	64.0	81	57.0	168	60.4	0.24 ^a^
Brothers/sisters	10	7.4	11	7.8	21	7.6	0.90 ^a^
Other	1	0.74	10	7.0	11	3.96	<0.01 ^a^
Current smoking	8	6.7	11	8.3	19	7.5	0.63 ^a^
Hazardous drinking	21	16.0	17	12.3	38	14.1	0.12 ^a^
	Mean	SD	Mean	SD	Mean	SD	
Age (years)	32.8	4.8	31.0	4.0	31.9	4.5	<0.01 ^b^
Individual Monthly Income (USD)	278.0	115.0	267.2	75.6	272.4	96.7	0.45 ^b^
Distance from homeland to the factory (km)			86.3	95.6			
Duration of migration (years)			2.1	3.0			

^a^ Chi-squared test; ^b^ Mann-whitney test.

**Table 2:** Working and social environment factors.

Characteristics	Non-Migrant		Migrant		Total		*p*-Value
	n	%	n	%	n	%	
Time for working with one posture							
Less than 30 min	35	29.9	49	37.4	84	33.9	0.43 ^a^
30–60 min	18	15.4	20	15.3	38	15.3	
More than 60 min	64	54.7	62	47.3	126	50.8	
Hazardous occupational exposures							
Noise	92	71.9	118	83.1	210	77.8	0.03 ^a^
Dust	72	56.3	65	45.8	137	50.7	0.09 ^a^
High temperature	81	63.3	77	54.2	158	58.5	0.13 ^a^
Toxic gas	48	37.5	54	38.0	102	37.8	0.93 ^a^
Poor lighting	11	8.6	13	9.2	24	8.9	0.87 ^a^
High humidity	15	11.7	10	7.0	25	9.3	0.19 ^a^
Toxic chemicals	50	39.1	48	33.8	98	36.3	0.37 ^a^
Accident prone working environmental	20	15.6	20	14.1	40	14.8	0.72 ^a^
Social support and community cohesion							
People in this area are willing to help each other	72	56.3	77	58.3	149	57.3	0.73 ^a^
People around living place are willing to help neighbors	72	56.3	72	54.6	144	55.4	0.78 ^a^
People live in harmony	74	57.8	73	55.3	147	56.5	0.68 ^a^
People around can be trusted and reliable	59	46.1	56	42.4	115	44.2	0.55 ^a^
People share the same value, life concept	16	12.5	24	18.2	40	15.4	0.20 ^a^
This area has a tremendous amount of trash	30	23.4	17	12.9	47	18.1	0.03 ^a^
This area has society’s vices	17	13.3	9	6.8	26	10.0	0.08 ^a^
This area has many fights and quarrels	7	5.5	1	0.8	8	3.1	0.03 ^a^
	Mean	SD	Mean	SD	Mean	SD	
Years of working	10.3	3.4	9.5	4.0	9.9	3.8	0.01 ^b^
Working hours per day	8.2	0.7	8.3	1.0	8.2	0.9	0.91 ^b^
Number of hazardous occupational exposures	2.8	2.1	2.8	2.0	2.8	2.1	0.91 ^b^
Social support score	4.5	2.1	4.7	2.1	4.6	2.1	0.71 ^b^

^a^ Chi-squared test; ^b^ Mann-whitney test.

**Table 3:** Health status, health-related quality of life and behaviors.

Characteristics	Non-Migrant		Migrant		Total		*p*-Value
	*n*	%	*n*	%	*n*	%	
EQ5D5L							
Having problems in mobility	34	26.8	52	37.4	86	32.3	0.06 ^a^
Having problems in self-care	2	1.6	7	5.1	9	3.4	0.18 ^a^
Having problems with usual activity	30	23.6	51	37.0	81	30.6	0.02 ^a^
Pain/Discomfort	66	52.4	92	69.2	158	61.0	0.01 ^a^
Anxiety/Depression	64	52.0	94	70.7	158	61.7	<0.01 ^a^
Number of health problems							
0	39	28.5	22	15.4	61	21.8	0.045 ^a^
1	36	26.3	44	30.8	80	28.6	
2	26	19.0	39	27.3	65	23.2	
>2	36	26.3	38	26.6	74	26.4	
	Mean	SD	Mean	SD	Mean	SD	
EQ-5D Index	0.78	0.23	0.69	0.19	0.74	0.21	<0.01 ^b^
Number of health problems	1.81	1.66	2.00	1.60	1.91	1.63	0.16 ^b^

^a^ Chi-squared test; ^b^ Mann-whitney test.

**Table 4:** Associated factors with health-related quality of life (HRQOL) and number of health problems.

Characteristic	EQ5D5L Index		Number of Health Problems	
	Coef.	95% CI	Coef.	95% CI
Migrant (Yes versus No)	−0.08 ***	−0.12; −0.03	0.20 **	0.001; 0.41
Gender (Female versus Male)	0.12 **	0.01; 0.23	0.08	−0.31; 0.48
Education (versus Under High school)				
High school	−0.04	−0.11; 0.02	0.27	−0.20; 0.74
Above High school	−0.07 *	−0.14; 0.01	0.32	−0.18; 0.81
Marital status (Having spouse/partner vs. Single)	0.04	−0.06; 0.14	0.37 *	−0.04; 0.78
Age	0.00	−0.01; 0.01	−0.01	−0.04; 0.03
Years of working	−0.00	−0.01; 0.001	0.02	−0.01; 0.05
Working hours per day	−0.02	−0.05; 0.01	0.01	−0.10; 0.12
Number of hazardous occupational exposures	−0.02 **	−0.03; −0.001	0.14 ***	0.09; 0.19
Time fixed a posture when working (Less than 30 min)				
30–60 min	−0.02	−0.09; 0.05	0.23	−0.10; 0.55
More than 60 min	−0.02	−0.07; 0.03	0.39 ***	0.15; 0.62
Number of health problems	−0.04 ***	−0.05; −0.02		
Social support (point)	0.00	−0.01; 0.01	−0.03	−0.08; 0.02
Hazardous drinking (Yes versus No)	0.07	−0.02; 0.17	−0.49 *	−1.03; 0.05
Current smoking (Yes versus No)	0.08	−0.04; 0.20	0.22	−0.21; 0.64

*** *p* < 0.01, ** *p* < 0.05, * *p* < 0.1.
